# Bioinformatics-driven design and protective efficacy assessment of a multi-epitope vaccine for *Toxoplasma gondii*

**DOI:** 10.3389/fimmu.2026.1830954

**Published:** 2026-07-31

**Authors:** Wenyong Feng, Lu Sun, Chenglong Yang, Jie Cao, Yongzhi Zhou, Jinlin Zhou, Longzheng Yu, Houshuang Zhang

**Affiliations:** 1 Key Laboratory of Animal Parasitology of Ministry of Agriculture, Shanghai Veterinary Research Institute, Chinese Academy of Agricultural Sciences, Shanghai, China; 2 College of Agriculture, Yanbian University, Yanji, Jilin, China

**Keywords:** bioinformatics, MEP1, multi-epitope vaccine, protective efficacy assessment, *Toxoplasma gondii*

## Abstract

**Introduction:**

*Toxoplasma gondii* can cause toxoplasmosis. It is an important type of pathogen within the broad category of emerging and re-emerging zoonoses. As an infectious disease featuring a complex multi-host transmission cycle, it poses an increasingly severe threat to global public health. No licensed vaccines are currently available for pets and humans, and thus a novel high-efficiency vaccine is urgently required.

**Methods:**

Six antigens (GRA1, MIC17A, OWP2, LEA880, LEA870, and a hypothetical protein LEA530) representing different stages of the parasite lifecycle were selected from ToxoDB. T-cell and B-cell epitopes were predicted using immunoinformatics tools and screened based on antigenicity, allergenicity, and toxicity. The multi-epitope peptide (MEP1) was evaluated using molecular docking with Toll-like receptor 4 (TLR4) and immune simulation. The optimized sequence was expressed in HEK293T cells as a recombinant plasmid (MEP1-pcDNA3.1) and further evaluated in BALB/c mice.

**Results:**

MEP1 contained 13 cytotoxic T lymphocyte epitopes, 16 helper T lymphocyte epitopes, and 12 B-cell epitopes, with a length of 732 amino acids and a predicted molecular weight of 75.73 kDa. The antigenicity score was 0.7343, and structural modeling indicated stable secondary and tertiary conformations. Molecular docking suggested strong binding affinity to TLR4. Immune simulation predicted increased B-cell and T-cell responses following vaccination. *In vivo*, MEP1-pcDNA3.1 immunization significantly increased serum IFN-γ levels (526.81 pg/mL) compared with PBS and pcDNA3.1 controls. Splenocyte proliferation was significantly enhanced in the MEP1-pcDNA3.1 group (SI = 1.58 ± 0.21) compared with PBS (1.10 ± 0.09) and pcDNA3.1 (1.12 ± 0.04) groups (*P* < 0.01). Following challenge with 5 × 10³ tachyzoites of the PLK strain, survival was markedly prolonged in vaccinated mice, whereas all control mice died within 2–4 days.

**Conclusion:**

This study demonstrates an immunoinformatics-guided multi-epitope vaccine strategy against *T. gondii*, supported by *in vivo* immunogenicity and partial protective efficacy in a mouse model.

## 1 Background

*Toxoplasma gondii* (*T. gondii*) is a protozoan parasite in the phylum *Apicomplexa*. It is an opportunistic, obligate parasite that primarily infects nucleated cells in warm-blooded animals. Humans can be infected by consuming food or water contaminated with sporulated oocysts shed by cats, which are definitive hosts, or by ingesting meat containing tissue cysts. Approximately one-third of the global human population is reported to be infected with *T. gondii*, although only a small proportion develops overt clinical symptoms (1, 2). When the organism parasitizes pregnant animals, it can cause fetal abortion and stillbirth, posing a severe threat to the livestock industry (3). With rising living standards, cats have become one of the most popular companion animals in many households. This has sharply increased the risk of toxoplasmosis in humans from *T. gondii* transmission between humans and cats. No licensed vaccine is currently available for humans. However, Toxovax^®^ vaccine has been used in some regions to prevent and control abortion in sheep caused by *T. gondii* (4).

The lifecycle of *T. gondii* is complex, with its sexual reproduction phase exclusively restricted to definitive hosts within the felid family. Upon ingesting tissue cysts from infected prey or sporulated oocysts from the environment, the parasite invades the intestinal epithelial cells of the cat, where it completes its sexual development (5, 6). The excreted unsporulated oocysts mature under suitable environmental conditions to form infectious sporulated oocysts, which serve as a major source of infection for humans and animals. In contrast, in other intermediate hosts, the parasite undergoes only asexual reproduction, producing infectious tissue cysts (7).

Beyond its well-recognized domestic transmission cycle, increasing evidence indicates that *T. gondii* is also maintained in wildlife populations through sylvatic transmission routes. This wildlife–livestock–human interface underscores its ecological adaptability and positions *T. gondii* as an important pathogen within the broader context of emerging and re-emerging zoonotic diseases. Consequently, infectious diseases with complex multi-host transmission cycles pose increasing threats to global health, requiring coordinated surveillance and control strategies under a unified One Health framework, integrating human, animal, and environmental health systems (8).

Several types of *Toxoplasma* vaccines are currently under development, including DNA vaccines, recombinant subunit vaccines, attenuated live vaccines, and mRNA vaccines. DNA vaccines are cost-effective, have a long half-life, and exhibit good biocompatibility, safety, and stability. However, they have relatively poor immunogenicity and fail to produce sufficient immune responses. Additional concerns include suboptimal bioavailability, potential immune tolerance, and allergic reactions (9). Recombinant subunit vaccines contain highly purified antigen proteins as the effective components and offer higher safety. However, these vaccines suffer from poor immunogenicity and require adjuvants to enhance their efficacy and prevent degradation by proteinases. Additionally, a single protein cannot induce antibodies against components of the entire lifecycle of *T. gondii* (10). Attenuated live vaccines can express a broader range of antigens at specific lifecycle stages, theoretically making them more efficient. However, their safety remains controversial, posing a major obstacle to clinical trials (11, 12). In recent years, mRNA vaccines have become a prominent focal point due to their rapid production timelines, safety, and the absence of risks associated with infection or insertional mutations. They can be rapidly taken up and expressed in the cytoplasm (13); however, mRNA susceptibility to extracellular RNase degradation necessitates advanced delivery systems, which increases production costs (13).

Bioinformatics-based approaches have emerged as powerful tools in modern vaccine development, particularly for the rational design of multi-epitope vaccines. Compared with traditional vaccine discovery methods, immunoinformatics enables rapid screening of antigenic epitopes with high precision, significantly reducing experimental time, cost, and labor intensity. In addition, computational prediction methods allow systematic evaluation of epitope antigenicity, immunogenicity, and MHC-binding affinity, facilitating the selection of vaccine candidates with improved immunological potential prior to experimental validation. Multi-epitope vaccines using highly conserved and immunogenic antigenic sites have become an effective strategy for developing new types of vaccines. Compared with traditional single-antigen vaccines, multi-epitope vaccines offer the advantage of incorporating freely selected specific epitopes. This design can elicit a multi-faceted and multi-layered immune response and is characterized by high stability and a lack of carcinogenic potential. Potential issues, such as allergenicity and toxicity, that may exist in the vaccine design process can be optimized using bioinformatics tools, thereby achieving high efficiency in vaccine preparation. However, multi-epitope vaccines share the disadvantage of low immunogenicity found in traditional subunit vaccines. This issue can be addressed by adding adjuvants or modifying the delivery system to enhance immunogenicity, potentially inducing strong and long-lasting humoral and cellular immunity, leading to more widespread and durable protection (14, 15).

Therefore, the objective of this study was to design a novel multi-epitope vaccine (MEP1) against *Toxoplasma gondii* using immunoinformatics approaches and to evaluate its immunogenicity and protective efficacy through *in vitro* and *in vivo* experiments in a BALB/c mouse model.

## 2 Materials and methods

### 2.1 Mice and ethics approval statement

The mice used in this study were 6 to 8-week-old female ICR or BALB/c mice purchased from Shanghai Jiesijie Laboratory Animal Co., Ltd., and housed at 50–60% humidity and 22 °C in the specific pathogen-free animal facility at the Shanghai Veterinary Research Institute, with free access to food and water. All procedures were approved by the Institutional Animal Care and Use Committee and the Animal Ethics Committee of the Shanghai Veterinary Research Institute (Ethics Protocol No. SV-20241227-10). Mice were euthanized by CO_2_ inhalation. Specifically, mice were placed in an euthanasia chamber, and CO_2_ was introduced at a flow rate of 10%–30% of the chamber volume per minute. After loss of consciousness, the flow rate was increased (not exceeding 0.5 kPa). Euthanasia was confirmed by respiratory arrest and dilated pupils, followed by maintaining CO_2_ exposure for an additional 2 minutes.

### 2.2 Cell culture and parasite

The *T. gondii* PLK strain, HFF cells, and HEK293T cells (human embryonic kidney cells) were stored in our laboratory. HFF cells were cultured in Dulbecco’s modified Eagle’s medium (DMEM) supplemented with streptomycin (100 mg/mL), penicillin (100 U/mL), and 5% fetal bovine serum (FBS) at 37 °C in a humidified 5% CO_2_ atmosphere. HEK293T cells were cultured under the same conditions except that the medium was supplemented with 10% FBS. *T. gondii* PLK strain tachyzoites were cultured in HFF cells using DMEM supplemented with 5% inactivated FBS.

### 2.3 Bioinformatics analysis

#### 2.3.1 Protein sequence retrieval

Transcriptomic data for *T. gondii* oocysts, tachyzoites, and bradyzoites were retrieved from the ToxoDB website (https://toxodb.org/toxo/app), expression profiles across developmental stages were visualized using RMA-normalized (log2) values, and genes exhibiting peak expression in sporulated oocysts were identified as candidate oocyst-enriched genes. Protein sequences corresponding to GRA1 (XM_002365660.2), MIC17A (XM_002371906.1), OWP2 (XM_018779474.1), LEA880 (XM_018781755), LEA870 (XM_002370394.1), and a hypothetical protein (LEA530) (XM_002369869.2) were obtained from the National Center for Biotechnology Information online database in FASTA format and saved for subsequent analysis. Multiple sequences of six target genes from representative *T. gondii* strains (RH, ME49, and PRU types I, II, and III) were aligned and screened using BLAST. Conservation analysis performed with MEGA11 software confirmed that each antigen exhibited over 90% sequence conservation.

#### 2.3.2 Epitope prediction

MHC class I and class II epitope predictions were performed using the H-2^d^ haplotype, which corresponds to the BALB/c mouse strain. B-cell epitopes for the aforementioned proteins were predicted using the online bioinformatics tool ABCPred (https://webs.iiitd.edu.in/raghava/abcpred/), with the epitope window length set to 16 amino acids, and the top two epitopes with the highest scores were selected for subsequent analysis (16, 17). Two online servers, IEDB (MHC I binding) (http://www.iedb.org/) and NetCTLpan 1.1 (https://services.healthtech.dtu.dk/services/NetCTLpan-1.1/), were used to predict cytotoxic T lymphocyte (CTL) epitopes. Overlapping candidate epitopes identified by both tools were retained (18–20). The epitope length set to 14 amino acids. Helper T cell (Th) epitopes were predicted via IEDB (MHC II binding) (http://www.iedb.org/) and NetMHCIIpan 4.0 (https://services.healthtech.dtu.dk/services/NetMHCIIpan-4.0/), and shared candidate epitopes from the two prediction results were screened for further use (18, 19, 21). The epitope length set to 15 amino acids. Other parameters of the above prediction tools were kept at default settings.

#### 2.3.3 Multi-epitope peptide design

The selected epitopes were curated by removing those located in signal peptide regions predicted by SignalP 4.1 (https://services.healthtech.dtu.dk/services/SignalP-4.1/) and merging duplicate sequences. The remaining epitopes were subsequently evaluated for antigenicity using VaxiJen v 2.0 (https://www.ddg-pharmfac.net/vaxijen/VaxiJen/VaxiJen.html). Epitopes achieving scores greater than 0.5 were selected as final candidate epitopes for multi-epitope peptide construction (22, 23). The top three CTL epitopes and Th epitopes were selected based on antigenicity prediction scores (maximum of three each), and the top two B-cell epitopes were chosen based on their respective scores. CTL epitopes were connected via AAY linkers, Th epitopes via GPGPG linkers, and B-cell epitopes via KK linkers. An EAAAK linker was incorporated at the N-terminus of the construct to connect the PADRE sequence. The full-length multi-epitope comprised 732 amino acid residues and ultimately formed the final multi-epitope peptide named MEP1.

#### 2.3.4 Prediction of homology, antigenicity, allergenicity, and physicochemical properties

To evaluate potential cross-reactivity and ensure sequence specificity, the full-length MEP1 amino acid sequence was analyzed using BLASTp against the NCBI non-redundant (nr) protein database (24). The antigenicity, toxicity, and allergenicity of MEP1 were analyzed using VaxiJen v2.0, ToxinPred (https://webs.iiitd.edu.in/raghava/toxinpred/index.html), and AllerTOP v.2.0 (https://www.ddg-pharmfac.net/AllerTOP/), respectively (22–26). The physicochemical properties of MEP1 were predicted using Expasy (https://web.expasy.org/protparam) (27).

#### 2.3.5 Prediction of secondary and tertiary structures

The secondary structure of MEP1 was analyzed using the SOPMA online server (http://npsa-prabi.ibcp.fr/cgi-bin/npsa_automat.pl?page=npsa_sopma.html) (28). The tertiary structure was predicted using AlphaFold software (29). The model was further validated by inspecting model’s Ramachandran plot through the platform SAVES v6.0 (https://saves.mbi.ucla.edu/) (30, 31). The design’s overall performance as seen in relation to the Z-value were evaluated using the ProSA website (https://prosa.services.came.sbg.ac.at/prosa.php) (32).

#### 2.3.6 Prediction of discontinuous B-cell epitopes and disulfide engineering analysis of MEP1

The discontinuous B-cell epitopes of MEP1 was analyzed using the Ellipro (https://tools.iedb.org/ellipro/). The disulfide engineering analysis was analyzed using the Disulfide by Design 2.0 (http://cptweb.cpt.wayne.edu/DbD2/) (33, 34). Keep all parameters as default.

#### 2.3.7 Molecular docking exploration with mouse toll-like receptor 4

We performed simulated docking between the designed MEP1 and murine Toll-like receptor 4 (TLR4) to evaluate their binding affinity. The protein structure of TLR4 was obtained from the Protein Data Bank under the unique identifier PDB ID 2Z64. Molecular docking was conducted using the web-based ClusPro tool (https://cluspro.org/home.php) (35–39). PyMOL software was subsequently used to visualize the three-dimensional structure of the MEP1-TLR4 complex and to render potential interaction sites. The PDBsum tool (https://www.ebi.ac.uk/thornton-srv/databases/pdbsum/) was used to identify the amino acid sequences mediating the interaction between MEP1 and TLR4, thereby facilitating a comprehensive understanding of their interaction sites and binding patterns (40).

#### 2.3.8 Simulation of immune responses

Immune responses triggered by MEP1 were simulated using the C-ImmSim online tool (https://150.146.2.1/C-IMMSIM/index.php?page=1) to evaluate the immunogenic efficacy of the MEP1 (41, 42). Modifications were applied to the simulation protocol, specifically with an administration regimen of 1000 vaccine molecules per injection, delivered at 2-week intervals for a total of three doses, while all other simulation parameters remained at their default settings.

#### 2.3.9 Codon optimization and *in silico* cloning

The amino acid sequence of MEP1 was submitted to Shanghai HuaGene Biotech Co., Ltd. for codon optimization and *de novo* gene synthesis. The optimized sequence was subsequently cloned into a pET-28a (+) expression vector. The recombinant plasmid was named MEP1- pET-28a (+).

### 2.4 *In vitro* assay

#### 2.4.1 Construction, verification, and transformation of the MEP1- pcDNA3.1 plasmid

The recombinant sequence (2196 bp) of the MEP1 gene with 6×His was amplified from MEP1- pET-28a (+) using PCR. The homologous recombination primers used were 5’-ccacactggactagtggatccATGGCTAAGTTCGTGGCAGC-3’ and 5’-cttggtaccgagctcggatccACACTTTTTTCGGGCGGC-3’, which contain a *Bam*H I restriction enzyme site. The PCR products of MEP1 were inserted into a pcDNA3.1 vector, and the recombinant MEP1-pcDNA3.1 plasmid. The recombinant plasmid was verified by DNA sequencing to ensure that all gene sequences were correct and then transformed into DH5α (DE3) competent cells. The specific procedures included adding the plasmid to thawed competent cells on ice, incubating on ice for 30 min, subjecting to 42 °C heat shock for 45 sec in a water bath, returning to ice for 2 min, adding antibiotic-free LB medium followed by 1 h incubation at 37 °C with 180 rpm shaking, and finally, plating onto ampicillin-containing agar plates.

#### 2.4.2 Preparation of MEP1-pcDNA3.1

Positive monoclonal colonies were inoculated and cultured overnight at 37 °C with 180 rpm shaking. Plasmid extraction was performed using an EndoFree Maxi Plasmid Kit (TIANGEN). Briefly, 200 mL bacterial culture was pelleted by centrifugation (8000 × g, 5 min, RT). The pellet was resuspended in 8 mL P1 buffer, mixed with 8 mL P2 buffer via 6–8 inversions, and incubated at RT for 5 min. After adding and mixing 8 mL P4 buffer until turbidity appeared, the lysate was centrifuged (8000 × g, 20 min), and the supernatant was retained. 0.3 volumes of isopropanol were mixed with the supernatant and loaded onto an adsorption column. Following centrifugation (8000 × g, 2 min), the column was washed twice with 10 mL PW wash buffer (2 min centrifugation each), then spun for an extra 5 min to remove ethanol residue. The column membrane was air-dried at RT, and plasmid DNA was eluted with 2 mL preheated (55 °C) TB buffer after 3 min RT incubation and 2 min centrifugation at 8000 × g. Purified plasmids were quantified and stored at −20 °C for further analysis.

#### 2.4.3 Transfection of HEK293T cells

HEK293T cells cultured in 6-well plates were transfected at 70–80% confluency. Culture medium was refreshed 2 h before transfection. Briefly, 2 μg MEP1-pcDNA3.1 plasmid was diluted in 50 μL serum-free DMEM, mixed with 4 μL PEI 40000 transfection reagent (Beijing LABLEAD Trading Co., Ltd.), and incubated at room temperature for 10–15 min to form transfection complexes. Complexes were added dropwise to cells with gentle plate swirling. Cells were harvested 48 h post-transfection for downstream assays.

#### 2.4.4 Western blot analysis

Harvested cells were washed three times with PBS and centrifuged at 3000 × g for 5 min at 4 °C to obtain cell pellets. Cells were lysed in 200 μL RIPA lysis buffer (Beyotime Biotechnology Co., Ltd.) for 30 min with vortexing every 5 min. After full lysis, samples were centrifuged at 12,000 × g for 10 min at 4 °C, and the resulting supernatants were collected as total protein extracts. Protein samples were mixed with an equal volume of 2× loading buffer and denatured at 100 °C for 10 min. Subsequently, proteins were separated by 12% SDS-PAGE and electrotransferred onto PVDF membranes via a semi-dry transfer system (25 V, 1 A, 20 min). Membranes were blocked with 5% skim milk at 37 °C for 1 h, followed by incubation with primary antibodies, including anti-His monoclonal antibody (1:10,000) or cat positive serum (1:1,000). HRP-conjugated goat anti-mouse IgG (1:5,000) and HRP-conjugated rabbit anti-cat IgG (1:5,000) were used as secondary antibodies, respectively. Protein bands were finally visualized using enhanced chemiluminescence reagent.

### 2.5 *In vivo* assay

#### 2.5.1 Preparation of *T. gondii* and lysate antigens

Tachyzoites were harvested by passing the culture preparation through a 27-gauge needle 3–5 times. The culture was filtered through a 5-μm filter to remove cellular debris. Tachyzoites were then centrifuged and washed three times with sterile PBS to remove cellular debris. The purified tachyzoites were disrupted by freezing at −80 °C and thawing at 37 °C three times, followed by sonication at 400 W for 5 min. Parasite lysate supernatant was harvested by centrifugation at 12, 000 g for 15 min at 4 °C. *T. gondii* lysate antigen (TLA) concentrations were measured using a BCA protein assay kit (Beyotime Biotechnology Co., Ltd).

#### 2.5.2 Measurement of antibody levels using enzyme-linked immunosorbent assays

Mouse tail venous blood was collected on day 14 after the third immunization to isolate serum samples. *T. gondii*-specific antibody levels were detected by ELISA following the manufacturer’s protocol. Briefly, 100 μL of TLA (20 μg/mL) was added to each well of a 96-well plate and incubated overnight at 4 °C. Then, the plate was blocked with 3% skim milk in PBS containing 0.05% Tween-20 (PBST) for 1 h at 37°C. The obtained serum samples were serially diluted with skim milk at specified ratios and used as the primary antibody. After three washes with PBST, the primary antibody was added to the plate, followed by incubation at 37°C for 1 h. After six washes with PBST, HRP-conjugated anti-mouse IgG (1:5000 diluted with 3% skim milk in PBST) was added to designated wells for 1 h at 37°C. Tetramethylbenzidine substrate solution was added for 15 min in the dark, the reaction was stopped with 2 M H_2_SO_4_, and the absorbance was read at OD_450_ nm using a microplate reader. Each sample was assayed in triplicate.

#### 2.5.3 Immunization, challenge, and determination of parasite loads

Thirty female BALB/c mice were randomly and equally divided into three groups (ten mice/group) using a computer-generated random number table and immunized with PBS (100 μL), pcDNA3.1 plasmid (100 μg), or MEP1-pcDNA3.1 (100 μg). On day 0 (primary immunization), day 14 (14 days after primary immunization), and day 28 (14 days after booster immunization) of the experiment, each mouse was intramuscularly administered either a solution containing 100 μg of plasmid DNA or an equivalent volume of PBS. For downstream analyses, three mice from each group were sacrificed for splenocyte proliferation assays, while the remaining seven mice per group were used for survival analysis following challenge infection.

Fourteen days after the third immunization, seven mice were intraperitoneally challenged with 5000 tachyzoites of the *T. gondii* PLK strain. Mouse survival was monitored daily, and moribund mice were humanely euthanized with CO. Heart, lung, spleen, brain, and liver tissues were collected to determine parasite loads via absolute qPCR. The qPCR assay was performed on an Applied Biosystems 7500 system using *T. gondii*-specific primers and probe (forward: 5’-GCTCGCCTGTGCTTGGAG-3’; reverse: 5’-ATCTTCTCCCTCTCCGACTCTC-3’; probe: FAM-TCGCTTCCCAACCACGCCACCC-BHQ1). Parasite abundance was quantified based on a standard curve generated with serially diluted *T. gondii* genomic DNA. All experiments were performed in triplicate.

#### 2.5.4 Cytokine assays

Serum cytokines at 14 days post the third immunization were quantified using a sandwich ELISA kit (Shanghai Yuli Biotech, Inc.). Briefly, standards were two-fold serially diluted, and wells were assigned for blanks, standards and samples. A total of 50 μL diluted standard was loaded into standard wells; sample wells received 40 μL diluent plus 10 μL serum (5-fold final dilution). Plates were sealed and incubated at 37 °C for 30 min, then washed five times (30 s per wash). All wells except blanks were supplemented with 50 μL enzyme conjugate, incubated again at 37 °C for 30 min, and washed five times. Reactions were terminated with stop solution, and OD450 values were read with blank-based zero calibration. Statistical comparisons were conducted via Student’s t-test.

#### 2.5.5 Lymphocyte proliferation assay

Three mice from each group were euthanized one day prior to *T. gondii* challenge. Under aseptic conditions, spleens were excised from three mice per group, then ground with a sterile grinding rod, and filtered through a nylon sieve. Splenic lymphocyte suspensions were collected, and red blood cells (RBCs) were removed using RBC lysis buffer (TIANGEN). After three washes with DMEM medium, splenic lymphocytes (10^5^ cells/well) were added to 96-well plates and cultured with TLA (20 µg/mL), 7.5 µg/mL concanavalin A (ConA) as a positive control, or DMEM medium alone (negative control) at 37 °C in 5% CO_2_ for 72 h. Cell proliferation was measured using a CCK-8 Cell Counting Kit (Vazyme) in accordance with the manufacturer’s instructions. Absorbance was measured at an OD of 450 nm.

#### 2.5.6 Statistical analysis

Statistical analyses were performed using SPSS Statistics software. Shapiro–Wilk normality test and Levene’s homogeneity of variance test were conducted prior to statistical comparison; all experimental data in this study conformed to normal distribution and exhibited homogeneous variances. All quantitative data were expressed as mean ± standard deviation (SD). One-way analysis of variance (ANOVA) followed by Tukey’s *post-hoc* multiple comparison test was adopted for multi-group comparisons involving antibody titers at serial dilutions, cytokine concentrations, and tissue-derived indicators from five different tissues. Two-group comparisons were performed using independent Student’s t-test. The Kaplan–Meier method combined with the log-rank test was utilized for survival curve analysis. A two-tailed p-value < 0.05 was defined as statistically significant.

## 3 Results

### 3.1 Design and construction of the multi-epitope peptide 1

**Figure 1:**
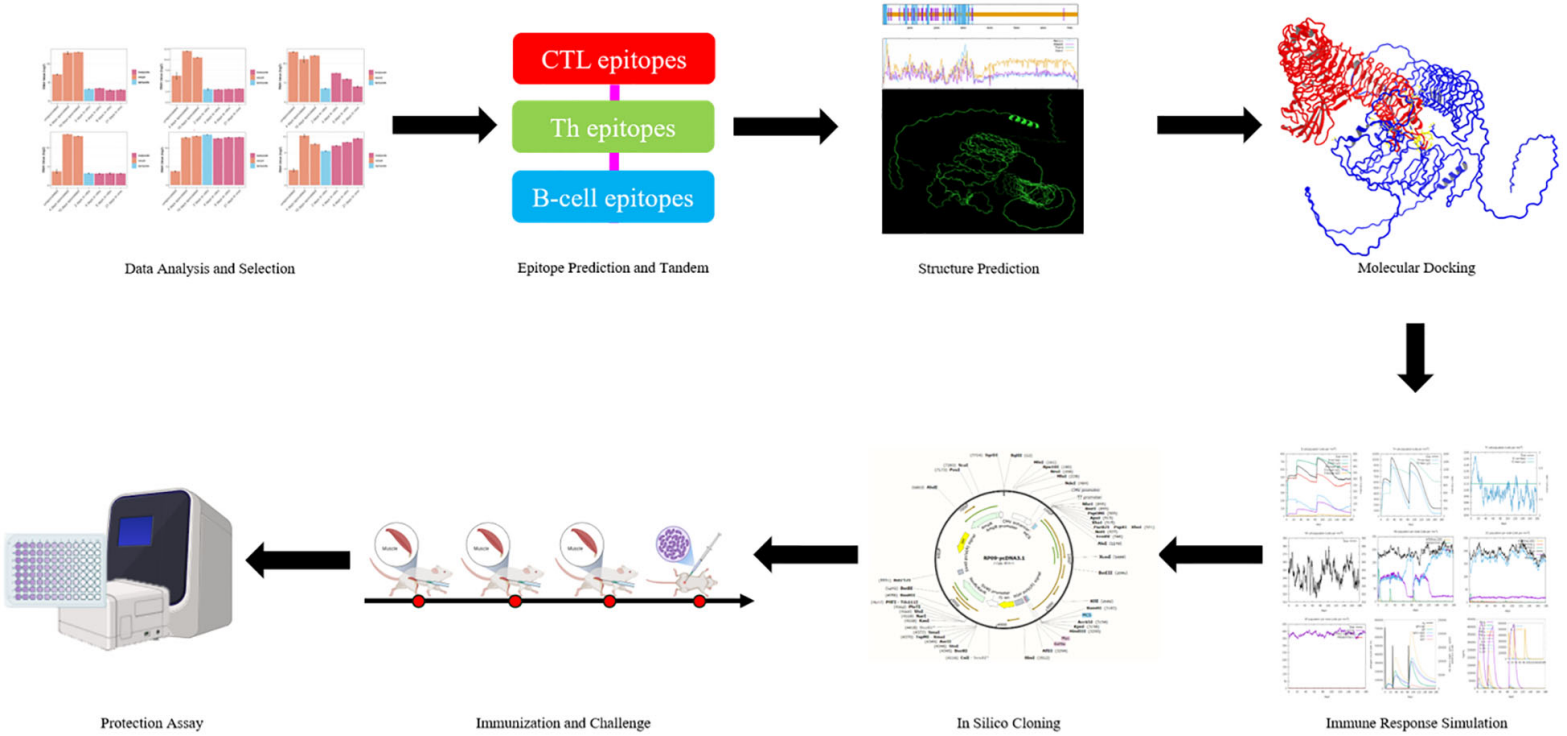
Overview of the research workflow. The bioinformatics analysis primarily encompassed gene screening, epitope prediction, structural prediction, molecular docking, and immune simulation. The successfully constructed nucleic acid vaccine was subsequently validated *in vitro* before being applied in *in vivo* experiments.

Bioinformatic tools predicted CTL, Th and B-cell epitopes from target proteins. Epitopes were screened by antigenicity scores, and validated non-toxic, non-allergenic epitopes are shown in **Table 1**. A total of 13 CTL epitopes, 16 Th-cell epitopes, and 12 B-cell epitopes were identified. The CTL epitopes were linked using AAY linkers, Th-cell epitopes were connected using GPGPG linkers, and B-cell epitopes were joined with KK linkers. An EAAAK linker was added to the N-terminus of the peptide to incorporate the universal MHC II epitope PADRE (AKFVAAWTLKAAA), thereby constructing the final multi-epitope peptide named MEP1. The constructed MEP1 comprised 732 amino acids. **Figure 1** shows the MEP1screening, construction, and experimental procedures.

**Table 1:** Antigenic epitope information for constructing multi-epitope peptides.

Antigen	Epitope type	Epitope sequence	Antigenicity	Allergenicity	Toxicity
TgGRA1	B-Cell	TSDGSYSEVGNVNMEE	0.9102	Non-allergen	Non-toxin
		EMMGNTYRVERPTGNP	0.7870	Non-allergen	Non-toxin
	CTL	KSMQRDEEI	1.0545	Non-allergen	Non-toxin
	Th	EGGDNQSSAVSDRAS	0.9507	Non-allergen	Non-toxin
		VCLSAGAYAAEGGDN	0.8086	Non-allergen	Non-toxin
TgMIC17A	B-Cell	RGETNMHKSGAPGLNT	0.7295	Non-allergen	Non-toxin
		PKHCGLPTTCVKERTK	0.6604	Non-allergen	Non-toxin
	CTL	NYDFRGETNM	1.8715	Non-allergen	Non-toxin
		EPKCKAFLF	1.7821	Non-allergen	Non-toxin
		AAEPKCKAFL	1.6308	Non-allergen	Non-toxin
	Th	KERTKYAGETVATFP	1.0438	Non-allergen	Non-toxin
		PSQENNNANAVSGPK	0.8828	Non-allergen	Non-toxin
		MKDIGYKGTDSKATK	0.8380	Non-allergen	Non-toxin
TgOWP2	B-Cell	PGEIVYECPVGFHCAS	0.7971	Non-allergen	Non-toxin
		SCLSIAPGEIVYECPV	0.7092	Non-allergen	Non-toxin
	CTL	APGEIVYEC	1.3844	Non-allergen	Non-toxin
		VYKIRKDSTI	0.5133	Non-allergen	Non-toxin
	Th	CPVGFHCASNAKNSD	0.7403	Non-allergen	Non-toxin
		EPEFICKEGQLINGN	0.6634	Non-allergen	Non-toxin
		KKNVVPTEVVDKDED	0.5476	Non-allergen	Non-toxin
LEA880	B-Cell	MSTASSAKEGVYDAGL	0.5783	Non-allergen	Non-toxin
		AGDYCSSAGMKVGDTA	0.5606	Non-allergen	Non-toxin
	CTL	VGDTASNAM	0.8266	Non-allergen	Non-toxin
	Th	GQKISDATKAARDAA	0.9313	Non-allergen	Non-toxin
		AAKEKTSSAVEEAGD	0.9014	Non-allergen	Non-toxin
LEA870	B-Cell	GEDAIMYGRELTGQTP	1.0679	Non-allergen	Non-toxin
		TPLIDSASSAGRQLKD	0.7218	Non-allergen	Non-toxin
	CTL	KHVTPKAAL	0.7264	Non-allergen	Non-toxin
		LSPPGSGDL	0.6898	Non-allergen	Non-toxin
		SASSAGRQL	0.5441	Non-allergen	Non-toxin
	Th	GRELTGQTPKEAAED	1.2661	Non-allergen	Non-toxin
		QAFVVSGMKAKDAAP	0.9006	Non-allergen	Non-toxin
		FAAKTRLTSVAQEAT	0.8062	Non-allergen	Non-toxin
LEA530	B-Cell	RRLGEEPDTEDELVVE	0.5448	Non-allergen	Non-toxin
		KGYWGGGYYRRLGEEP	0.5241	Non-allergen	Non-toxin
	CTL	KTPVTKVTYL	0.8413	Non-allergen	Non-toxin
		RSEPKKSVSY	0.7633	Non-allergen	Non-toxin
		YQPKKTVRTV	0.7476	Non-allergen	Non-toxin
	Th	PEELQAQEPEESADE	0.9796	Non-allergen	Non-toxin
		PKKLIRSEPKKSVSY	0.8060	Non-allergen	Non-toxin
		QPSYTTKVVRRPKKV	0.7765	Non-allergen	Non-toxin

### 3.2 Prediction of homology, antigenicity, allergenicity, and physicochemical properties

BLAST results from NCBI revealed that its homology with murine and human proteins was less than 20%, indicating a low risk of molecular mimicry. The evaluation results indicated that MEP1 is non-toxic and non-allergenic, with an antigenicity score of 0.7343. The physicochemical properties of MEP1 were assessed using Expasy. MEP1 was found to comprise 732 amino acids, including 86 negatively charged residues and 102 positively charged residues, with a molecular weight of 75.73 kDa. The prediction results showed that the theoretical isoelectric point (pI) of MEP1 was 8.92, and it contained 10,534 atoms. Its chemical formula is represented as C_3297_H_5218_N_930_O_1063_S_26_. The predicted half-life of MEP1 was 4.4 hours *in vitro*, more than 20 hours in yeast (*in vivo*), and more than 10 hours in *E. coli* (*in vivo*). The instability index of the protein was calculated to be 33.19, classifying it as stable. Notably, the aliphatic index of the protein was 49.60, indicating relatively high thermostability. Furthermore, the grand average of hydropathicity (GRAVY) value of MEP1 was −0.728, demonstrating high hydrophilicity.

### 3.3 Prediction of secondary and tertiary structures

**Figure 2:**
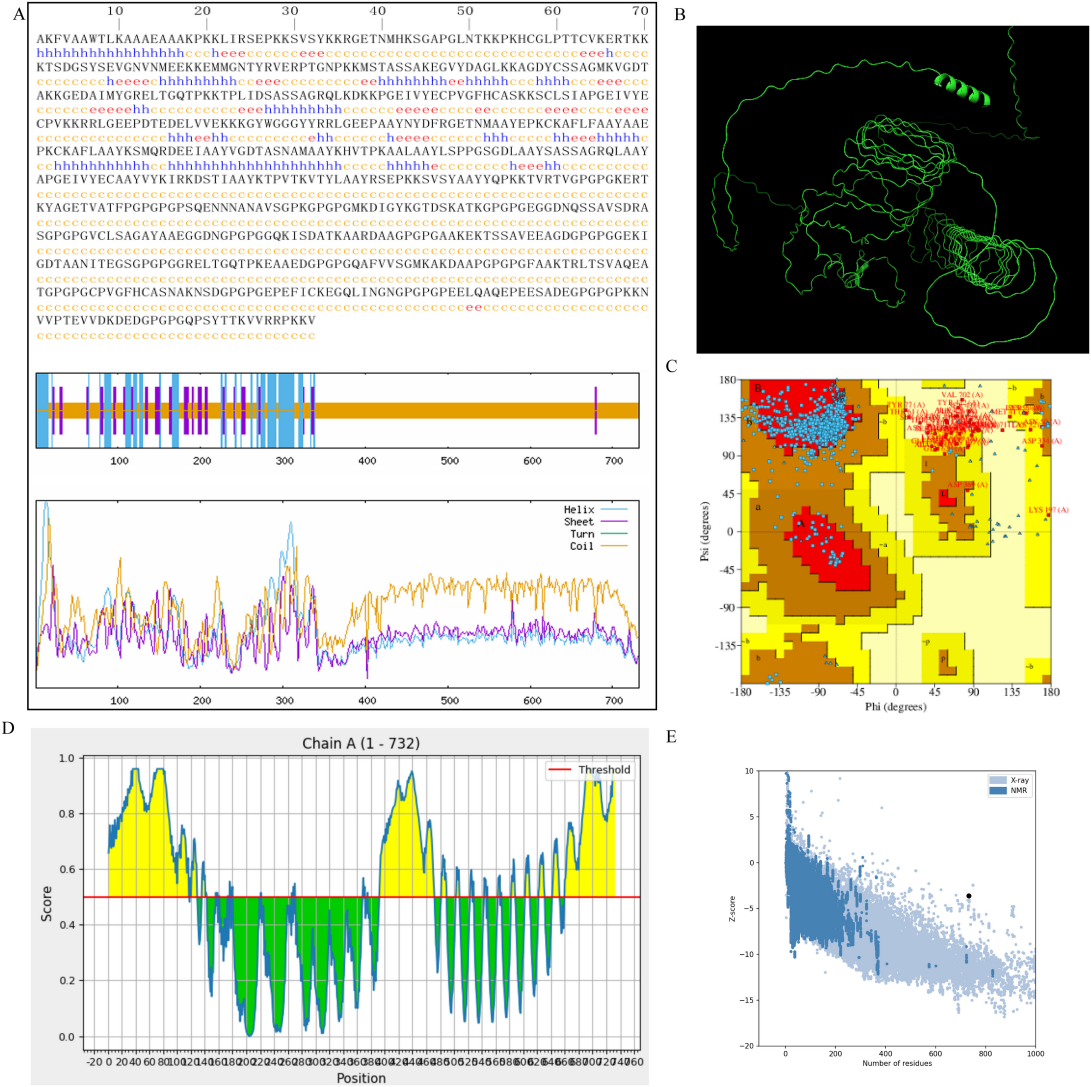
Secondary and three-dimensional structure prediction and quality assessment. **(A)** Secondary structure prediction of the MEP1 sequence using the SOPMA online program. **(B)** Three-dimensional structure of MEP1 predicted using AlphaFold software. **(C)** Ramachandran plot of MEP1. **(D)** Prediction of discontinuous B-cell epitopes. **(E)** Z-score evaluation of the MEP1 structural model.

The secondary structure of MEP1, consisting of 732 amino acids, was analyzed using the SOPMA online program. The prediction results revealed that MEP1 comprised 15.57% α-helices, 8.47% β-sheets, and 75.96% random coils (**Figure 2A**). This distribution indicates the predominance of random coils in the peptide’s secondary structure, with α-helices and β-sheets occupying relatively minor proportions.

The tertiary structure of MEP1 was analyzed using AlphaFold software, and the initial peptide model was visualized through PyMOL (**Figure 2B**). Analysis of the Ramachandran plot for the constructed MEP1 demonstrated that over 90% of its amino acid residues reside in allowed regions, confirming the high reliability of this tertiary structural model (**Figure 2C**). To further assess structural reliability, the ProSA-web server was used, yielding a Z-score of -3.62, which falls within the range of experimentally determined protein structures of similar size, indicating a native-like overall folding energy profile (**Figure 2E**).

### 3.4 Prediction of discontinuous B-cell epitopes and disulfide engineering analysis of MEP1

Conformational B-cell epitope prediction using the ElliPro server indicated multiple high-scoring discontinuous epitope regions distributed across the MEP1 structure. Notably, the N-terminal, central loop, and C-terminal regions exhibited scores above the threshold (0.5), suggesting potential surface-accessible antibody-binding sites (**Figure 2D**). The ElliPro prediction results revealed that MEP1 contains 35 discontinuous epitopes consisting of a total of 350 amino acid residues, with a maximum score of 0.930. The detailed scoring data are presented in **Table 2**. Disulfide engineering analysis using the Disulfide by Design 2.0 server identified several residue pairs with favorable geometric and energetic properties for potential disulfide bond formation. Among these, GLU425–THR426, SER73–ASP74, and TYR77–SER78 exhibited optimal energy scores (0.35–2.08 kcal/mol) and acceptable χ3 torsion angles, suggesting their suitability for stabilizing the MEP1 construct through site-directed cysteine substitution.

**Table 2:** ElliPro-based prediction of conformational B-cell epitopes of MEP1.

No.	Predicted residues	Number of residues	ElliPro score
1	E687, S688, A689, D690, E691, G692, P693, G694, P695, G696, P697, K698	12	0.93
2	K71, T72, S73, D74, G75, S76, Y77, S78, E79, V80, G81, N82, V83, N84, M85, E86, E87	17	0.929
3	R36, G37, E38, T39, N40, M41, H42, K43, S44, G45, A46, P47, G48, L49, N50, T51, K52	17	0.924
4	R728, P729, K730	3	0.919
5	K29, S30, V31, S32, Y33	5	0.883
6	K699, N700, V701, V702, P703, T704, E705, V706, V707, D708, K709, D710, E711, D712, G713, P714, G715, P716, G717, Q718, P719, S720, Y721	23	0.875
7	K53, P54, K55, H56, C57, G58, L59, P60, T61, T62, C63, V64, K65, E66, R67, T68, K69	17	0.863
8	T410, V411, G412, P413, G414, P415, G416, K417, E418, R419, T420, K421, Y422, A423, G424, E425, T426, V427, A428, T429, A430, P431, G432, P433, G434, P435, G436, P437, S438, Q439, E440, N441, N442, N443, A444, N445, A446, V447, S448, G449, P450, K451, G452, P453, A454, P455, G456, A457	48	0.84
9	R24, S25, E26, P27, K28	5	0.838
10	T722, T723, K724	3	0.817
11	K406, T407, V408	3	0.776
12	E684, P685, E686	3	0.772
13	K10, A11, A12, A13, E14, A15, A16, A17, K18, P19, K20	11	0.762
14	Q403, P404, K405	3	0.747
15	D459, I460, G461, Y462	4	0.707
16	E664, G665, Q666, L667, I668, N669, G670, N671, G672, P673, G674, P675, G676, E678	15	0.7
17	A1, K2, V4, A5, A6, T8, L9	7	0.688
18	E679, Q681, A682, Q683	4	0.684
19	P101, T102, G103, N104, P105, K106, K107, M108, S109, T110, A111, S112, S113, A114, K115	15	0.682
20	K463, G464, T465, D466, S467, K468, A469, T470, K471, G472	10	0.675
21	M92, G93, N94, T95, A96	5	0.663
22	E391, P392, K393, K394, S395, V396, S397, Y398, A399, A400, Y401	11	0.641
23	R97, V98, E99	3	0.628
24	F660, I661, C662, K663	4	0.603
25	F641, H642, C643, A644, S645, N646, A647, K648, N649, S650, D651, G652, P653	13	0.603
26	N481, S483, S484, A485, V486, S487, D488, R489	8	0.579
27	S602, G603, M604, K605, A606, K607, D608, A609, A610	9	0.569
28	D120, A121, G122, L123, K124, K125, A126, G127, D128, Y129, G134, M135, K136, V137, G138, D139, T140, A141, A142, E145	20	0.566
29	T621, L623, T624, S625, V626, A627, Q628, E629, A630, T631, G632	11	0.559
30	G582, Q583, T584, P585, K586, E587, A588, A589, E590	9	0.529
31	A523, T524, K525, A526, A527, R528, D529	7	0.527
32	A501, G502, A503, Y504, A505, A506, E507, G508	8	0.525
33	S543, S544, V545, V546, E547	5	0.517
34	T563, A564, A565, N566, I567, T568, E569	7	0.516
35	K368, D369, S370, T371, I372	5	0.508

### 3.5 Molecular docking with mouse toll-tike receptor 4

**Figure 3:**
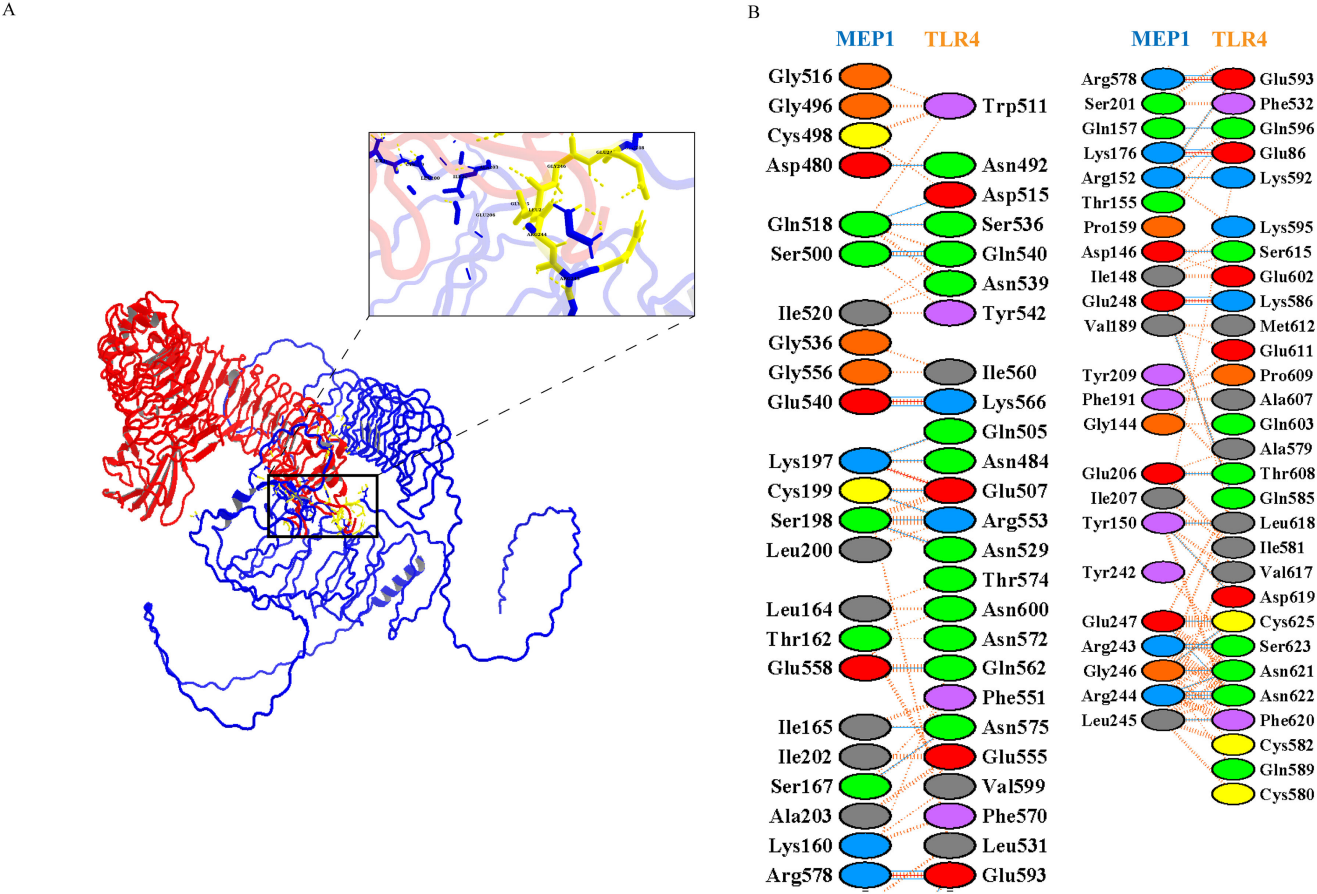
Molecular docking of the designed MEP1 with mouse toll-like receptor 4. **(A)** MEP1-TLR4 docked complex. **(B)** Residue interaction between the designed MEP1 and TLR4. Molecular interaction study of the docked complex using PDBsum, where red indicates salt bridges, yellow indicates disulfide bonds, blue indicates hydrogen bonds, and orange indicates non-bonded contacts.

MEP1 was computationally docked with TLR4 using the ClusPro web server. Cluster scores were ranked in the “Balanced” mode, and the result with a weighted energy score of −1273.4 was selected as the optimal model. This scoring mode calculates model scores using a specific formula, effectively reflecting the interactions between the receptor and the ligand. The obtained interaction model was visualized using PyMOL (**Figure 3A**). MEP1-TLR4 interactions were investigated by analyzing the binding strength of amino acid residues involved in receptor-ligand interactions using the PDBsum tool. In the MEP1-TLR4 complex, MEP1 comprises 45 interface residues, while TLR4 has 53 interface residues, with interface areas of 2723 Å^2^ and 2546 Å^2^, respectively. The complex formed five salt bridges, 41 hydrogen bonds, and 415 non-bonded contacts, suggesting potential recognition by TLR4 (**Figure 3B**).

### 3.6 Immune response simulation

**Figure 4:**
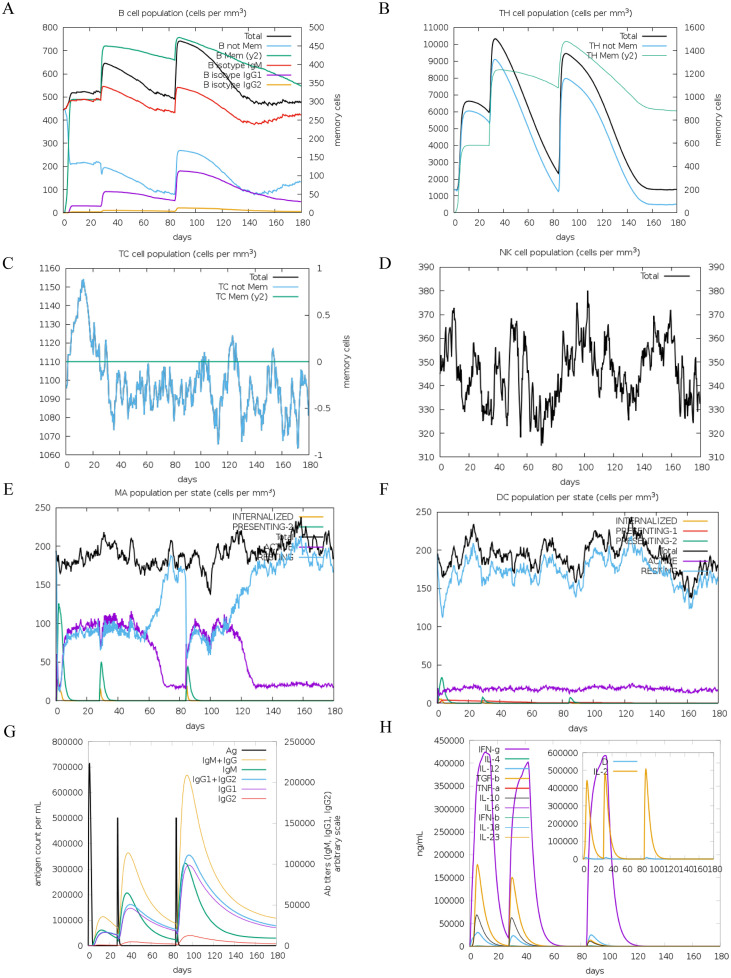
Immune simulation in silico. **(A)** B-lymphocyte count after three MEP1 doses. **(B)** Total T-lymphocyte count after three MEP1 doses. **(C)** Cytotoxic T lymphocyte (CTL) count after three MEP1 doses. **(D)** Natural killer (NK) cell counts after three MEP1 doses. **(E)** Macrophage counts in different states. **(F)** Dendritic cell counts in different states. **(G)** Antigen and antibody levels following vaccination. **(H)** Concentrations of various cytokines and interleukins after immunization.

Immune simulation serves as a critical reference for predicting whether a peptide can induce an effective immune response. Analysis of the simulation results revealed that antibody titers increased significantly with successive immunizations, indicating that MEP1 could induce a humoral immune response (**Figure 4**). Furthermore, the secondary immune response was significantly enhanced compared with the primary immune response, particularly B-lymphocyte (**Figure 4A**), Th (**Figure 4B**), CTL (**Figure 4C**), natural killer (NK) cell (**Figure 4D**), and macrophage (**Figure 4E**) activity. Additionally, elevated concentrations of dendritic cells suggested efficient antigen presentation by these antigen-presenting cells (**Figure 4F**). Finally, cytokine and interleukin levels were markedly elevated after immunization. These results indicate that MEP1 can stimulate high levels of antibodies (**Figure 4G**) and cytokines (**Figure 4H**), effectively activate immune cells and antigen-presenting cells, and facilitate their functions, thus demonstrating its strong immunogenicity and suggesting its potential to provide high-level protection against pathogenic infections.

### 3.7 PCR identification of MEP1-pcDNA3.1

**Figure 5:**
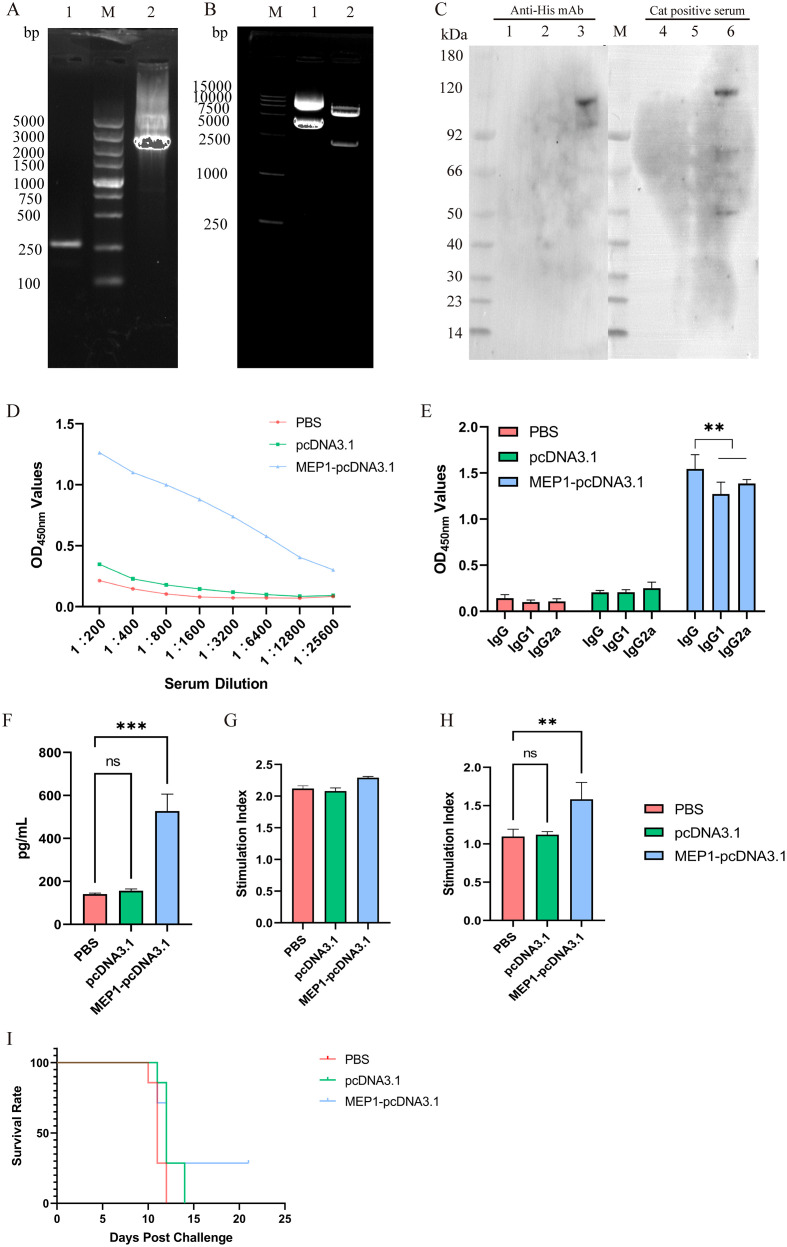
Construction of the recombinant plasmid and evaluation of immunoprotection. **(A)** Amplification of the MEP1 gene. M, DL 5000 DNA marker. Lane 1, pcDNA3.1 plasmid. Lane 2, MEP1-pcDNA3.1 plasmid. **(B)** Verification of recombinant plasmid by double-enzyme restriction digestion. M, DL 1,5000 DNA marker. Lane 1, control plasmid DNA. Lane 2, digested plasmid DNA. **(C)** Western blot analysis of the MEP1-pcDNA3.1 expressed protein. M, protein marker. Lanes 1 and 4, HEK293T cell lysates. Lanes 2 and 5, lysates of HEK293T cells transfected with pcDNA3.1. Lanes 3 and 6, lysates of HEK293T cells transfected with MEP1-pcDNA3.1. Left panel (lanes 1–3): samples probed with anti-His monoclonal antibody. Right panel (lanes 4–6): samples probed with cat positive serum. **(D)** Determination of antibody titers. **(E)** Determination of antibody isotypes. **(F)** Serum IFN-γ levels. **(G, H)** Splenic lymphocyte proliferation. After stimulation with either ConA **(G)** or TLA **(H)**, the stimulation index (SI) was calculated as the ratio of the OD450 of stimulated cells to the OD450 of unstimulated cells. **(I)** Survival curves of immunized mice after *T. gondii* challenge. *P < 0.05, **P < 0.01, and ***P < 0.001.

MEP1 with approximately 2242 bp was successfully amplified using the plasmid MEP1-pET28a (+) containing the synthesized MEP1 fragment as a template. The DNA fragment of the correct size was gel-purified and subsequently ligated into the pcDNA3.1 eukaryotic expression vector. Monoclonal colonies were screened using universal primers, yielding a PCR product of approximately 2600 bp (**Figure 5A**). The bacterial cultures corresponding to PCR-positive clones were sent for sequencing, which confirmed 100% sequence identity, thereby obtaining the desired positive plasmid.

### 3.8 Western blot analysis of MEP1-pcDNA3.1 expression

The collected cells were lysed using RIPA lysis buffer and centrifuged. The supernatant and pellet were then separated for western blot analysis to assess protein expression. A single band at approximately 95 kDa was detected (**Figure 5C**), which was larger than the expected molecular weight.

### 3.9 Determination of antibody levels using ELISA

The following experiments were conducted using biological replicates. The ELISA results demonstrated that a high level of antibodies was induced in the MEP1-pcDNA3.1 group (**Figure 5D**). The total IgG level in the MEP1 group was significantly higher than that in the PBS and pcDNA3.1 control groups (*P* < 0.05). Additionally, we evaluated the levels of IgG1 and IgG2a antibody subtypes. In the MEP1-pcDNA3.1 group, the IgG2a levels (2.115 ± 0.020) were higher than the IgG1 levels (1.806 ± 0.025) (*P* < 0.01), indicating that MEP1-pcDNA3.1 predominantly elicits a Th1-type cellular immune response (**Figure 5E**).

### 3.10 Cytokine assays

The following experiments were conducted using biological replicates. The MEP1-pcDNA3.1 group showed significantly increased serum levels of IFN-γ compared with the PBS and pcDNA3.1 control groups. As a key cytokine in controlling *T. gondii* infection, IFN-γ concentrations reached 526.81 ± 79.68 pg/mL, which was markedly higher than that in the control group (*P* < 0.05) (**Figure 5F**).

### 3.11 Splenocyte proliferation assay

The following experiments were conducted using biological replicates. Fourteen days after the final immunization, spleens were collected from three mice per group. Splenocytes were stimulated with ConA (**Figure 5G**) or TLA (**Figure 5H**). Lymphocyte proliferative capacity was assessed using the Cell Counting Kit-8 (CCK-8). The stimulation index (SI) value was 1.10 ± 0.09 in the PBS group, 1.12 ± 0.04 in the pcDNA3.1 control group, and 1.58 ± 0.21 in the MEP1-pcDNA3.1 group. Splenocyte proliferation in the MEP1-pcDNA3.1 group was significantly higher than that in either the PBS group or the pcDNA3.1 control group (*P* < 0.01).

### 3.12 Survival curve

Fourteen days after the last immunization, seven mice per group were challenged with 5×10^3^ tachyzoites of the *T. gondii* PLK strain, and survival was recorded daily. Mice in two control groups began to die on day 10, and all succumbed within 2–4 days (**Figure 5I**). In contrast, survival was prolonged in the MEP1-pcDNA3.1 group compared with the control group, with deaths first occurring on day 11.

### 3.13 Parasite load assay

**Figure 6:**
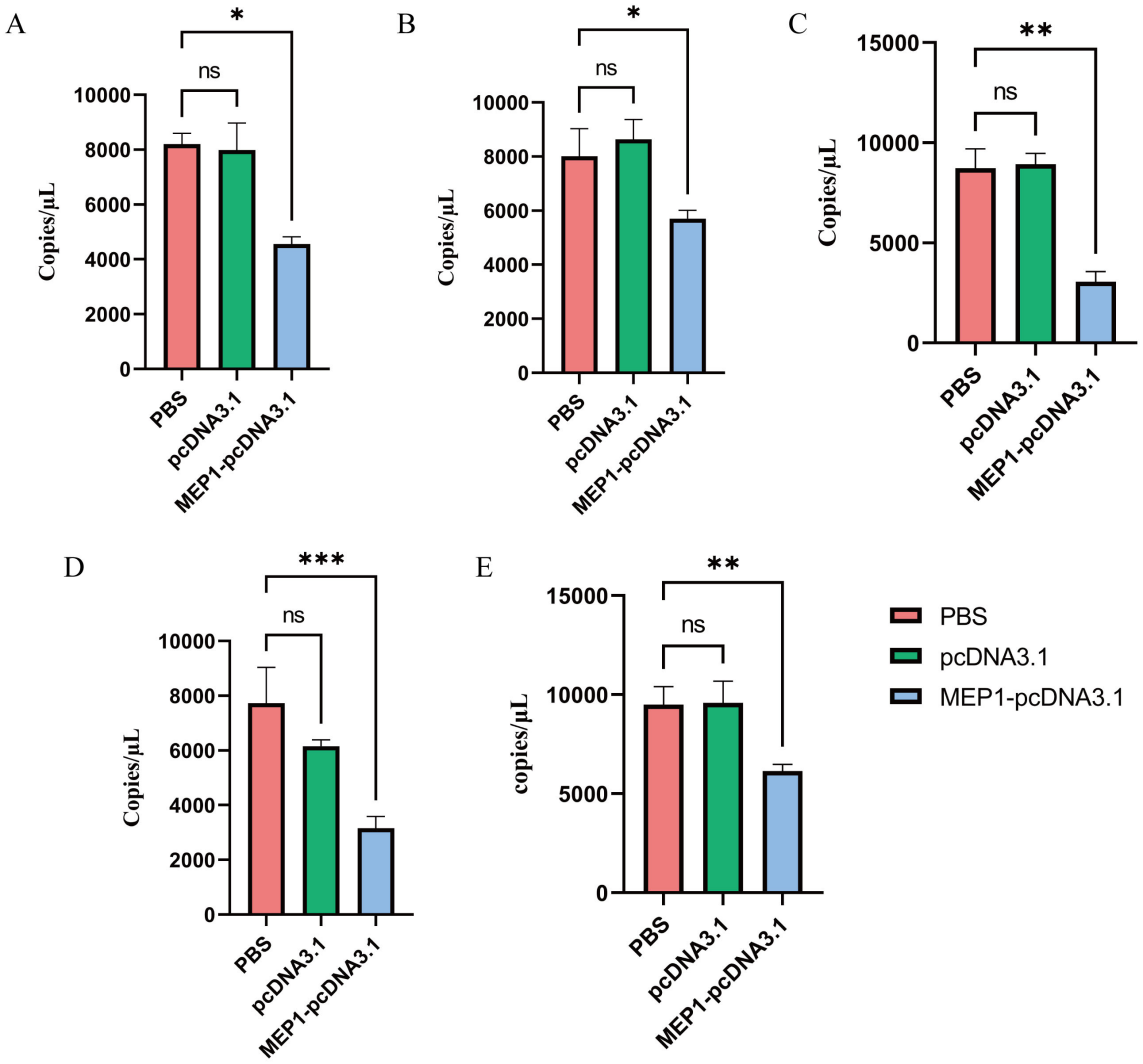
Parasite loads in different tissues. **(A)** Heart, **(B)** lung, **(C)** liver, **(D)** spleen, and **(E)** brain. Data are presented as means ± SD of three independent experiments. *P < 0.05, **P < 0.01, and ***P < 0.001.

The following experiments were conducted using biological replicates. Parasite load in mouse organs (heart, liver, spleen, lung, and brain) from the three groups was analyzed by real-time quantitative PCR. Mice from each group showing clear symptoms of toxoplasmosis but still alive were selected. After euthanasia by CO_2_ inhalation and their heart, liver, spleen, lung, and brain tissues were collected to assess parasite load. Mice in the MEP1-pcDNA3.1 group showed a significant decrease in parasite numbers in the heart (*P* < 0.05), liver (*P* < 0.05), spleen (*P* < 0.05), lung (*P* < 0.01), and brain (*P* < 0.01) (**Figure 6**) compared with mice in the PBS and pcDNA3.1 control groups, indicating a marked decrease in parasite load in all examined tissues.

## 4 Discussion

*Toxoplasma gondii*, a representative zoonotic protozoan pathogen categorized among emerging zoonotic diseases (EZDs), is a globally prevalent intracellular apicomplexan parasite. The felid family is the only definitive host, while nearly all warm-blooded animals act as intermediate hosts, and increases in domestic cat ownership have facilitated increased transmission to humans. This establishing a complete transmission cycle linking human populations, livestock, wildlife and ecological environments. It stands out as a high-priority food-borne zoonosis that requires integrated intervention under the unified “One Health” framework as highlighted in the review (8). *T. gondii* infection can lead to severe neurological disorders in immunocompromised individuals and spontaneous abortion in pregnant women, posing a serious threat to human and animal health (43). Although current drug therapies can moderately control acute infection, their efficacy in eliminating tissue cysts remains limited and is often accompanied by toxicity concerns. Consequently, existing treatments fail to achieve complete pathogen eradication or fully resolve infection-related sequelae.

Various types of vaccines are currently considered the most effective tool against *T. gondii* infection, including live-attenuated vaccine (43–46), inactivated vaccine (47–49), DNA vaccine (4, 50), and recombinant protein vaccine (15, 50–53). Recombinant immunogenic proteins play a significant role in toxoplasmosis prevention research. However, *T. gondii* expresses numerous critical proteins throughout its lifecycle that are involved in invasion, proliferation, and oocyst formation, making it difficult for single-protein vaccines to elicit sufficient immune protection. Distinctions in certain antigenic epitopes are also present in different strain types. The development of multi-epitope vaccines using highly conserved and immunogenic antigenic epitopes has emerged as a novel strategy for toxoplasmosis vaccine development. The protective efficacy of existing candidate vaccines varies widely, and no traditional subunit vaccine has been able to provide complete protection (52, 53). Unlike conventional single-protein subunit vaccines, multi-epitope vaccines incorporate multiple selectable epitopes capable of inducing comprehensive and multi-layered immune protection against different stages of the *T. gondii* lifecycle, offer improved stability, and lack carcinogenic potential.

In the context of advancing artificial intelligence (AI), bioinformatics tools provide crucial support for designing efficient and safe multi-epitope vaccines. Multi-epitope vaccines integrate specific antigenic epitopes to simultaneously stimulate cellular and humoral immunity while avoiding potential immune side effects associated with full-pathogen protein (54). Immuno-informatics approaches have allowed the successful design of multi-epitope vaccines for a range of pathogens, including *Mycobacterium tuberculosis* and SARS-CoV-2. However, most multi-epitope vaccines are still limited to in silico studies and have not been verified by *in vitro* or *in vivo* experiments (36, 55). Importantly, this study moves beyond in silico prediction by integrating computationally identified cytotoxic T lymphocyte (CTL), helper T lymphocyte (Th), and B cell epitopes into a rationally designed multi-epitope construct (MEP1) and further validating its immunogenic potential using a DNA vaccine platform in a BALB/c mouse model. This represents a critical transition from purely predictive vaccine design toward experimental immunological validation in a biologically relevant *in vivo* system.

The sexual reproduction of *T. gondii* occurs within the intestinal tract of its definitive host. The resulting oocysts, after maturing under suitable environmental conditions, become a major source of transmission to humans and livestock. Therefore, inhibiting or even blocking oocyst production is important for controlling toxoplasmosis. In our MEP1 design process, we selected the antigens GRA1 and MIC17A from the ToxoDB database. These antigens are expressed during both acute and chronic infection stages and have demonstrated good protective efficacy. Despite their high predicted antigenicity, their application in a multi-epitope chimeric vaccine has not been reported (56, 57). We additionally integrated transcriptomic data with existing research to identify four proteins, LEA880, LEA870, OWP2, and a hypothetical protein (LEA530), which are highly expressed during the oocyst stage, to use in a vaccine to prevent *T. gondii* infection in feline hosts and block transmission at its source. Our preliminary experiments indicated their promising protective potential. The inclusion of these oocyst-stage antigens aims to reduce the quantity or viability of oocysts formed within the definitive host (58, 59). The six selected antigens were chosen based on their biological roles across different stages of *Toxoplasma gondii* development and their potential immunological relevance. GRA and MIC family proteins are known to be secreted or associated with parasite-host interaction interfaces, making them accessible to the host immune system. In addition, these proteins have been previously reported to exhibit immunogenic properties and are considered promising vaccine candidates in toxoplasmosis research (56–58). Sequence conservation across different strains further supports their suitability as stable targets for vaccine design, reducing the risk of immune escape. Therefore, the inclusion of these antigens provides both biological relevance and immunological coverage across multiple stages of parasite infection.

The construction of the multi-epitope peptide required identifying MHC class I and MHC class II epitopes, which play crucial roles in T lymphocyte recognition. B-cell epitopes responsible for humoral immunity are equally important, as they can further inhibit and eliminate the pathogen. All antigenic epitopes meeting the screening criteria were compiled, and those with high antigenicity scores, non-toxicity, and non-allergenicity, specifically CTL, Th, and B-cell epitopes, were selected as the foundation for vaccine assembly. We employed specific linkers to rationally assemble the epitopes and enhance the immunogenic potency of MEP1. The AAY linker was used to connect CTL epitopes, as it facilitates intracellular proteasomal cleavage and TAP transporter-mediated delivery, thereby enhancing MHC class I antigen presentation. The GPGPG linker was used to join Th epitopes because of its capacity to reduce conformational flexibility and promote efficient epitope processing and presentation. The KK linker was applied to connect B-cell epitopes as it can guide proteases for precise cleavage, helping to ensure that the resulting antibodies recognize the correct native conformation (54, 56, 60). Finally, the universal MHC class II epitope PADRE (AKFVAAWTLKAAA) can be processed by antigen-presenting cells, presented on the cell surface in complex with MHC class II molecules, and subsequently recognized by CD4+ T cells, particularly Th cells (61). From a biological feasibility perspective, the incorporation of linker sequences plays a critical role in minimizing junctional immunogenicity and preventing the formation of neo-epitopes, thereby maintaining epitope integrity during antigen processing. The presence of an N-terminal adjuvant further supports potential engagement with pattern recognition receptors, thereby improving the likelihood of immune system activation. Structural modeling of the full-length construct suggests that the vaccine adopts a stable tertiary conformation, with predicted surface exposure of major antigenic epitopes, which is essential for effective B-cell recognition and antigen accessibility. In addition, physicochemical property analysis indicates that the construct exhibits acceptable stability and molecular characteristics compatible with recombinant expression systems.

An optimal model typically boasts a higher concentration of residues located in the Ramachandran-favored regions and additional allowed regions, along with a lower proportion in the generously allowed regions and disallowed regions. B-cell epitope prediction is an essential component in the rational design of multi-epitope vaccines because antibody recognition is frequently dependent on the three-dimensional conformation of antigenic regions rather than only the linear amino acid sequence. Although linear B-cell epitope prediction provides preliminary information regarding potential antibody-binding regions, discontinuous epitopes formed by spatially adjacent but sequentially separated residues often represent the major determinants recognized by antibodies. Therefore, conformational B-cell epitope analysis was additionally performed in this study using the predicted three-dimensional structure of MEP1. The structural stability of engineered multi-epitope vaccines represents an important factor affecting their biological performance, as flexible or unstable protein conformations may compromise epitope presentation and immune recognition. In this study, disulfide engineering analysis was performed using the Disulfide by Design 2.0 server to identify potential residue pairs suitable for rational disulfide bond introduction. Engineered disulfide bonds can increase conformational rigidity, reduce excessive structural flexibility, and potentially improve the stability of recombinant vaccine antigens. Although these predicted modifications require further experimental validation, the disulfide engineering analysis provides additional structural support for the rational optimization of the MEP1 vaccine candidate.

We analyzed the binding mode and dynamic stability of MEP1 with the TLR4 receptor using molecular docking studies to elucidate the molecular interactions between peptide components and host immune receptors. The results demonstrated high affinity and stable binding conformations between MEP1 and TLR4, indicating its potential to effectively initiate innate immune signaling pathways and providing a theoretical basis for eliciting adaptive immune responses. Although molecular docking and interaction analyses indicated a stable and favorable binding between MEP1 and TLR4, these results parameters primarily reflect structural complementarity and binding propensity, rather than functional receptor activation. In the case of TLR4, immune activation requires not only ligand binding but also conformational rearrangement of the TLR4–MD2 complex and subsequent receptor dimerization, which ultimately triggers downstream signaling pathways such as NF-κB activation and cytokine production. Therefore, our findings suggest that MEP1 may be capable of being recognized by TLR4 as a potential ligand, supporting its immunological relevance, but do not directly demonstrate immune activation. Further immune simulation predictions revealed that vaccination could induce high antibody titers, activate B and T cells, and increase key cytokine levels, suggesting that the constructed MEP1 might activate immune responses via TLR4-mediated immune pathways and elicit potent immune responses. These responses collectively indicate an effective activation of both the non-specific and specific arms of the immune system, thereby enhancing the host’s capacity to eliminate the pathogen. Moreover, codon optimization and *in vitro* expression prediction performed in an *E. coli* system demonstrated that the peptide sequence exhibited a high adaptation index and optimal GC content, suggesting its strong potential for efficient expression in prokaryotic systems. These results support the feasibility of subsequent recombinant protein production and the development of other forms of vaccines.

The *in vitro* results demonstrated that the MEP1-pcDNA3.1 was successfully expressed in HEK293T cells. It is noteworthy that the observed molecular weight of MEP1 (~95 kDa) in SDS-PAGE/Western blot was higher than its theoretically predicted molecular weight (~75.73 kDa). Such discrepancies between predicted and apparent molecular weights have been widely reported for recombinant and chimeric proteins. SDS-PAGE determines the apparent molecular weight, which may deviate from the theoretical value due to differences in amino acid composition, protein conformation, SDS-binding efficiency, and electrophoretic mobility. In particular, multi-epitope or artificially designed fusion proteins often exhibit anomalous migration behavior on SDS-PAGE gels, leading to apparent molecular weights that differ from their calculated sizes. Therefore, the 95 kDa band observed in this study likely represents the apparent molecular weight of eukaryotically expressed MEP1 protein under denaturing electrophoretic conditions rather than its exact molecular mass. To further validate the precise molecular weight and post-translational status of the expressed protein, mass spectrometry-based analysis is currently underway. Further *in vivo* experiments revealed that MEP1-pcDNA3.1 could induce immune protection in the host, accompanied by a significant increase in *T. gondii*-specific IFN-γ, which plays a critical role in anti-*Toxoplasma* immunity. The survival rate of MEP1-pcDNA3.1-immunized mice was also significantly improved; however, it did not reach 100%. This limitation may be attributed to multiple factors, including the parasite challenge dose, immunization dosage, vaccine type, and other related variables. The multi-epitope peptide designed in this study demonstrated promising immunogenicity *in silico*. However, its practical application may still face certain challenges. In this study, TLA was adopted to detect humoral and cellular immune responses triggered by MEP1-pcDNA3.1 vaccination, a widely accepted evaluation approach for multi-epitope vaccines against *T. gondii*. Nevertheless, we recognize that immune reactivity against total parasite lysate only mirrors the global anti-*T. gondii* immune status instead of exclusive MEP1 epitope-specific immunity. Although we have successfully prepared purified recombinant MEP1 protein and anti-MEP1 polyclonal antibodies in our laboratory and verified specific binding between vaccine-induced serum and recombinant MEP1 via preliminary immunoassays, the complete datasets of recombinant MEP1-based ELISA and splenocyte restimulation were not incorporated into the current manuscript due to the preset scope and timeline of this work. Therefore, the present work cannot fully delineate epitope-specific immune signatures targeting MEP1. This limitation should be noted when interpreting our immune detection results. The elevated antibody levels and enhanced splenocyte activation observed herein principally demonstrate that the MEP1-pcDNA3.1 vaccine elicits protective immunity against *T. gondii* challenge, consistent with mainstream evaluation standards for epitope vaccine research using whole-parasite antigens. Follow-up research will implement immunoassays coated with recombinant MEP1 protein and synthetic MEP1 peptides to precisely dissect MEP1-specific humoral and cellular immune responses, and the corresponding comprehensive data will be reported separately. Although a series of *in silico* analyses predicted favorable potential to activate both innate and adaptive immune responses, consistency between computational predictions and actual experimental outcomes remains to be fully validated (62). In general, key characteristics, such as epitope conservatism, MHC binding capacity, and physicochemical properties, predicted by AI models often show good agreement with experimental results, providing reliable clues for antigen screening and vaccine design. In contrast, inconsistencies frequently exist in indicators closely related to the *in vivo* microenvironment, including actual expression efficiency, protein folding state, real immunogenicity in animal models, the duration of immune protection, and interference from host individual differences or immune tolerance (63, 64).

Therefore, while AI-assisted design greatly improves the efficiency and rationality of vaccine development, *in vitro* and *in vivo* experiments remain indispensable in verifying actual immune effects and bridging the gap between computational prediction and practical applications. In a future study, we will employ bioinformatics tools to further optimize the structure of the multi-epitope peptide and use computational approaches, such as molecular dynamics simulations, to assess the stability of the MEP1-TLR4 complex in a simulated environment, thereby validating the reliability of the static docking results. A follow-up study is currently being conducted to rigorously assess whether the MEP1 nucleic acid vaccine confers superior immunogenicity relative to independently administered single-antigen nucleic acid constructs. Future work will also focus on the *in vitro* expression and purification of MEP1, as well as the investigation of antibody and cellular immune responses in mice immunized with vaccines using other novel delivery systems, and also be conducted to evaluate the effect of different vaccine types on immunogenicity.

## 5 Conclusion

In the present study, a multi-epitope peptide (MEP1) was successfully constructed using an *in silico* design based on six antigens from distinct stages of the *T. gondii* lifecycle. Bioinformatics predictions demonstrated that MEP1 exhibited favorable stability, high solubility, and strong antigenicity and immunogenicity, with binding affinity to TLR4. Subsequent *in vitro* and *in vivo* experiments verified that MEP1-pcDNA3.1 could be expressed in cells and that MEP1-pcDNA3.1 significantly elicited specific immune responses *in vivo*. Notably, MEP1-pcDNA3.1 demonstrated protective efficacy against *T. gondii* infection by prolonging the survival time of infected mice and reducing parasitic burden in tissues, which was highly consistent with the bioinformatics predictions.

## Data Availability

The original contributions presented in the study are included in the article/supplementary material, further inquiries can be directed to the corresponding authors.
